# Boosting of enzymatic softwood saccharification by fungal GH5 and GH26 endomannanases

**DOI:** 10.1186/s13068-018-1184-y

**Published:** 2018-07-17

**Authors:** Pernille von Freiesleben, Nikolaj Spodsberg, Anne Stenbæk, Henrik Stålbrand, Kristian B. R. M. Krogh, Anne S. Meyer

**Affiliations:** 1 Novozymes A/S, Krogshøjvej 36, 2880 Bagsværd, Denmark; 2 Protein Chemistry & Enzyme Technology, DTU Bioengineering, Technical University of Denmark, Building 221, 2800 Kgs. Lyngby, Denmark; 3 Department of Biochemistry and Structural Biology, Center for Molecular Protein Science, Lund University, PO Box 124, 221 00 Lund, Sweden

**Keywords:** Glucose release, Endo-*β*(1→4)-mannanases, GH5, GH26, CBM1 (carbohydrate-binding module 1), CBM35, Galactoglucomannan, Acetylation

## Abstract

**Background:**

Softwood is a promising feedstock for lignocellulosic biorefineries, but as it contains galactoglucomannan efficient mannan-degrading enzymes are required to unlock its full potential.

**Results:**

Boosting of the saccharification of pretreated softwood (Canadian lodgepole pine) was investigated for 10 fungal endo-*β*(1→4)-mannanases (endomannanases) from GH5 and GH26, including 6 novel GH26 enzymes. The endomannanases from *Trichoderma reesei (Tres*Man5A) and *Podospora anserina* (*Pans*Man26) were investigated with and without their carbohydrate-binding module (CBM). The pH optimum and initial rates of enzyme catalysed hydrolysis were determined on pure *β*-mannans, including acetylated and deacetylated spruce galactoglucomannan. Melting temperature (Tm) and stability of the endomannanases during prolonged incubations were also assessed. The highest initial rates on the pure mannans were attained by GH26 endomannanases. Acetylation tended to decrease the enzymatic rates to different extents depending on the enzyme. Despite exhibiting low rates on the pure mannan substrates, *Tres*Man5A with CBM1 catalysed highest release among the endomannanases of both mannose and glucose during softwood saccharification. The presence of the CBM1 as well as the catalytic capability of the *Tres*Man5A core module itself seemed to allow fast and more profound degradation of portions of the mannan that led to better cellulose degradation. In contrast, the presence of the CBM35 did not change the performance of *Pans*Man26 in softwood saccharification.

**Conclusions:**

This study identified *Tres*Man5A as the best endomannanase for increasing cellulase catalysed glucose release from softwood. Except for the superior performance of *Tres*Man5A, the fungal GH5 and GH26 endomannanases generally performed on par on the lignocellulosic matrix. The work also illustrated the importance of using genuine lignocellulosic substrates rather than simple model substrates when selecting enzymes for industrial biomass applications.

**Electronic supplementary material:**

The online version of this article (10.1186/s13068-018-1184-y) contains supplementary material, which is available to authorized users.

## Background

**Fig. 1:**
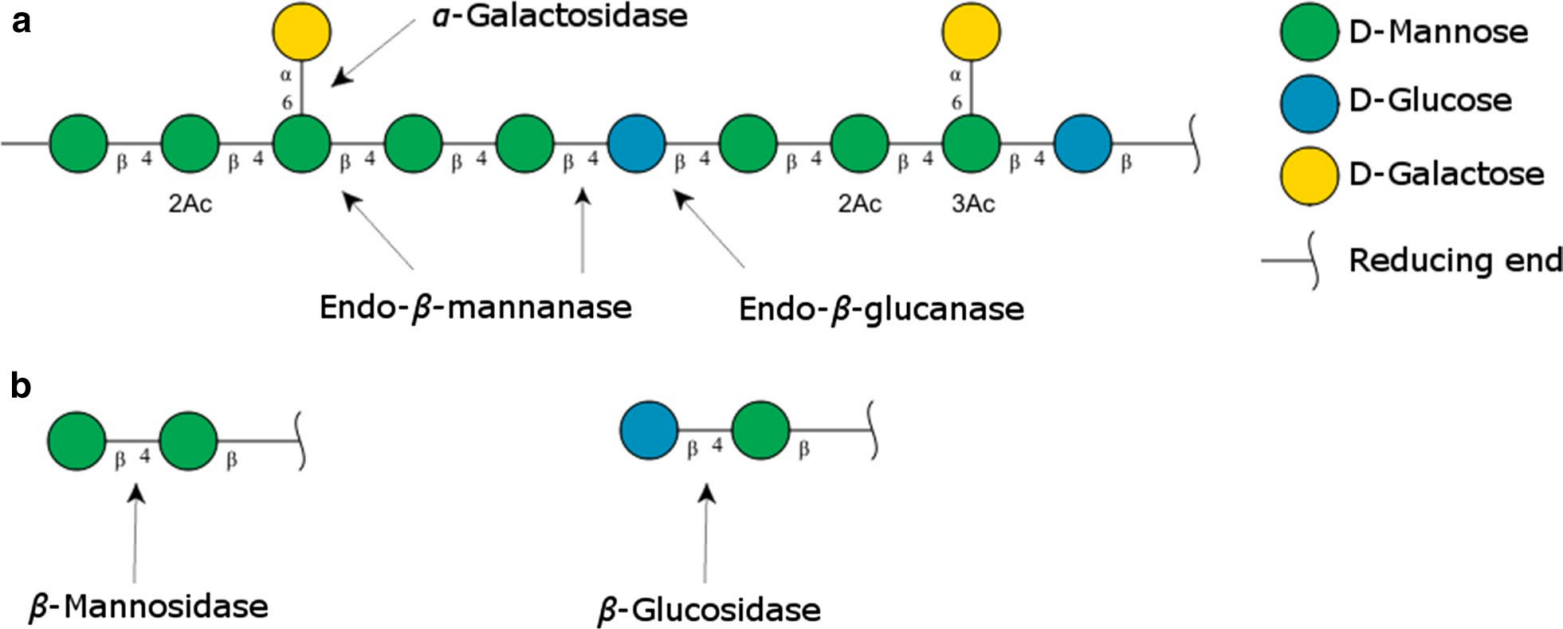
Schematic illustration of *O*-acetylated galactoglucomannan and enzymes involved in degradation of the backbone (**a**) and released oligosaccharides (**b**). Sugars shown using the Consortium for Functional Glycomics notation [48]. The figure shows general glycoside linkage specificity of each type of enzyme and a given enzyme may be restricted by neighbouring backbone sugar monomers and/or substitutions, exemplified by the varying influence of galactosyl substituents and potentially backbone glucosyl units on mannanase activity [17, 26]. Dual linkage specificity is known to occur among some of the illustrated enzymes. As example, some endo-*β*(1→4)-glucanases may hydrolyse within the glucomannan backbones, either by action on the glucopyranosyl units or by being unspecific, i.e., hydrolysing at mannopyranosyl units [16]

Softwood has significant potential as feedstock for renewable energy production and biorefining, due to its abundancy, low cost, and lack of competition with the food and feed industry. Enzymatic degradation of softwood to fermentable monomeric sugars is, however, still challenging due to its complex composition and inhomogeneous architecture [1]. Not only lignin but also hemicellulose, *β*-1,4 mannan and *β*-1,4 xylan (hereafter named mannan and xylan), prevents enzymatic hydrolysis of cellulose in the absence of relevant accessory enzymes [2, 3]. The hemicelluloses are closely associated with the cellulose fibrils and with lignin [1, 3–5]. The main hemicellulose in softwood is *O*-acetylated galactoglucomannan (Fig. 1), accounting for 15–25% of the wood dry matter [6, 7]. Galactoglucomannan consists of a *β*-1,4 linked backbone of d-mannopyranosyl and d-glucopyranosyl units. The mannopyranosyl units can be decorated with single *α*-1,6 linked d-galactopyranosyl residues at C-6 and be *O*-acetylated at C-2 and C-3 [6, 8]. The typical Man:Glc:Gal ratio in Norway spruce galactoglucomannan has been reported to be 3.5–4.5:1:0.5–1.1 with the mannopyranosyl residues being *O*-acetylated to an approximate degree of 0.2–0.3 [9–11]. Variations in the ratios depend on the raw material, but also extraction methods and pretreatment can reduce the amount of backbone decorations [11]. Mannans are not only found as structural units in plant cell walls, but also serve as storage polysaccharides in certain species, e.g., guar gum from the seeds of the guar plant (*Cyamopsis tetragonolobus*, Man:Gal, 2:1), locust bean gum, from the carob tree (*Ceratonia siliqua,* Man:Gal, 4:1) and the glucomannan from the konjac plant (*Amorphophallus konjac,* Man:Glc, 1.6:1) [7, 12].

It requires a coordinated interplay of different types of enzymes to degrade the *O*-acetylated galactoglucomannan found in softwood (Fig. 1). A variety of bacteria, yeasts and filamentous fungi express these mannan-degrading enzymes [13]: endo-*β*(1→4)-mannanases (endomannanases, EC 3.2.1.78), *β*-mannosidases (EC 3.2.1.25), *β*-glucosidases (EC 3.2.1.21), *α*-galactosidases (EC 3.2.1.22) and acetyl mannan or glucomannan esterases (EC 3.1.1.-) [14, 15]. Also, certain *β*(1→4)-glucanases (EC 3.2.1.4), primarily attacking the cellulose fraction of the softwood, have been shown to have activity on glucomannans [16].

Endomannanases are important enzymes for facilitating the solubilization and release of mannan from the substrate matrix [3, 17]. Endomannanases are classified into four glycosyl hydrolase (GH) families: 5, 26, 113 and 134 based on sequence similarity [18]. The endomannanases from family 5, 26, and 113, all belonging to clan GH-A, share a (*β*/*α*)_8_-TIM barrel fold in their structure, and catalyse the cleavage of the *O*-glycosidic bonds with retention of the anomeric configuration [19–21]. Based on studies of bacterial *Cellvibrio* mannanases, it has been proposed that GH26 enzymes may primarily attack soluble mannans, whilst the GH5 counterparts primarily attack insoluble mannans [22, 23]. However, it is unclear whether this perception is valid for the fungal GH26 endomannanases [24]. Regarding fungal endomannanases, different substrate binding modes were observed for the two *Podospora anserina* endomannanases, *Pans*Man26A and *Pans*Man5A [24, 25]. The *Pans*Man26A together with the GH26 endomannanase from *Aspergillus nidulans*, *Anid*Man26A, were also found to accommodate more galactopyranosyl residues in the active site pocket than their GH5 counterparts [26].

Many fungal GH5 endomannanases are modular, typically having a carbohydrate-binding module from family 1 (CBM1) as part of their structure. CBM1 is known to confer cellulose binding and increase the mannan hydrolysis of complex substrates such as softwood and ivory nut extractions containing both mannan and cellulose [27, 28]. Fungal GH26 endomannanases may have a CBM35 [24, 29], a CBM family known to bind to *β*-mannans, uronic acids and α-d-galactopyranosyl residues on carbohydrate polymers [30, 31].

The capacity of endomannanases to boost saccharification of softwood to fermentable monomers has been demonstrated and studied mostly with selected fungal GH5 endomannanases. The available literature in particular includes several studies with the *Trichoderma reesei* GH5 endomannanase, (*Tres*Man5A) [3, 4, 17, 32], but also of other endomannanases [33]. A few studies have shown increased glucose release from wood substrates when cellulase (and xylanase) cocktails have been supplemented with fungal GH26 endomannanases [29, 34], but a comparison of the performance of several different endomannanases on the same softwood substrate is not available in the literature. Severe pretreatment methods, leaving only small amounts of mannan in the pretreated substrate, are generally used on softwood to allow enzymatic saccharification. However, as the quest for efficient, yet sustainable utilization of plant biomass increases, new tailor-made pretreatment methods that also maximize the hemicellulose recovery, including mannan recovery, have appeared [35].

Based on the hypothesis that fungal endomannanases differ in their capacity to catalyse removal of galactoglucomannans from cellulose fibrils, and thus in turn may have different effects on enzymatic cellulose saccharification, this study compares 10 fungal endomannanases and their boosting effect on enzymatic cellulose degradation from softwood (the softwood being pretreated lodgepole pine*, Pinus contorta*) with 12% mannan left after pretreatment. The saccharification studies were performed at low temperature (30 °C) to focus on comparing activity of the enzymes, and at 50 °C to mimic industrial saccharification conditions. A subsidiary aim was to address the importance of the CBM35 in softwood saccharification by testing the *Podospora anserina* GH26 endomannanase with and without its CBM35. To touch upon any possible differences in the biological role of the fungal GH5 and GH26 endomannanases, and to assess if any of the substrate preferences on pure mannans could help explain performance differences on the softwood substrate, the initial rate of the studied endomannanases on soluble mannans, including acetylated and deacetylated spruce galactoglucomannan were also determined.

## Results and discussion

Based on a phylogenetic sequence comparison of more than 50 fungal GH26 endomannanases, and subsequent recombinant expression assessment, 8 wild type ascomycete GH26 endomannanases were selected for investigation, 6 of them previously uncharacterised (Table 1). Two of the GH26 endomannanases carry both a N-terminal CBM35, a common module among fungal GH26 enzymes [24, 29], as well as a C-terminal CBM1, previously only found in fungal GH5 endomannanases. In addition, two previously characterized GH5 endomannanases from *A. niger* [36] and *T. reesei* [27], respectively, were included in the study. The selected enzymes are all expressed well in the fungal host *Aspergillus oryzae.* The enzymes were all expressed using their native gene sequence and natural signal peptide and purified from the culture broth, the latter indicating that they function as secreted enzymes in nature. To address the influence of their CBMs, the *P. anserina* GH26 and *T. reesei* GH5 enzymes were expressed both with and without their CBM, i.e., CBM35 and CBM1, respectively (Table 1).

**Table 1:** Properties of the studied endomannanases

Origin	Domains	Mw^a^	pH_opt_^b^	Relative activity	Tm^c^	*t*½^d^	Sequence ID^e^	Identity^f^
		kDa		pH_5_/pH_opt_	°C	h		%
GH26								
*Collariella virescens* (*Cvir*Man26A)	CBM35-GH26-CBM1	57.9	6 (5–7)	0.97	62	–	BBW45415	76
*Mycothermus thermophiles* (*Mthe*Man26A)	CBM35-GH26	52.1	5 (5–8)	1.00	68	91 ± 0.3	MH208368	76
*Podospora anserina* (PansMan26A)	CBM35-GH26	49.8	6 (5–7)	0.97	57	90 ± 5.5	B2AEP0, [24]	100
*Podospora anserina* (*Pans*Man26A core)	GH26	34.4	5 (5–7)	1.00	58	103 ± 2.2	(B2AEP0)	100
*Neoascochyta desmazieri* (*Ndes*Man26A)	CBM35-GH26	48.7	5 (4–7)	1.00	65	267 ± 24.1	MH208367	60
*Westerdykella* sp. (*Wsp.*Man26A)	CBM35-GH26	50.4	6 (6–7)	0.79	58	59 ± 4.3	MH208369	55
*Ascobolus stictoideus* (*Asti*Man26A)	CBM35-GH26-CBM1	59.4	7 (5–7)	0.80	61	81 ± 7.7	BBW45412	55
*Aspergillus nidulans* (*Anid*Man26A)	GH26	35.2	6 (5–7)	0.93	53	10 ± 0.1	Q5AWB7, [26]	52
*Yunnania penicillata* (*Ypen*Man26A)	GH26	34.5	6 (5–8)	0.87	50	21 ± 0.1	BDN98740	48
GH5								
*Trichoderma reesei* (*Tres*Man5A)	GH5-CBM1	45.2	4 (4–5)	0.93	81	–	Q99036, [27]	36
*Trichoderma reesei* (*Tres*Man5A core)	GH5	38.8	4 (3–6)	0.88	78	2390 ± 360	(Q99036), [27]	36
*Aspergillus niger* (*Anig*Man5A)	GH5	39.8	4 (3–5)	0.85	87	137 ± 10.0	BCK48306, [36]	30

^a^Theoretical (non-glycosylated protein)

^b^pH optimum at 37 °C and pH interval with 80% relative activity in brackets

^c^The thermal midpoint (Tm) at pH 5

^d^Half-lives (*t*½) at 30 °C. No decay was observed for *Cvir*Man26A and *Tres*Man5A during the 48 h incubation period (Additional file 1: Figure S1)

^e^A reference is given when the enzyme is previously characterized

^f^Homology to *Pans*Man26A for which the structure is known (PDB ID: 3ZM8)

### Physicochemical properties of the enzymes

The GH26 endomannanases had pH optima in the range of 5–7 and Tm between 50 and 68 °C, with the two wild type core enzymes, *Anid*Man26A and *Ypen*Man26A, having lowest Tm of 53 and 50 °C, respectively (Table 1). Despite the high Tm values, *Ypen*Man26A and *Anid*Man26A had surprisingly low half-lives during prolonged incubation at 30 °C (Table 1). Tm is considered the temperature at which the protein molecule unfolds. However, according to the classical van’t Hoff equation and the Arrhenius equation, the equilibrium constant for protein denaturation and the rate of the enzymatic reaction vary with temperature. In practice, this implies that inactivation and rate constant changes caused by altered conformation of enzymes may occur (gradually and slowly) at lower temperatures than the Tm. The net effect is that altered conformation of the enzyme proteins may cause gradual activity loss during prolonged incubation as can be seen in the 30 °C stability data. The GH5 endomannanases had pH optima in the range of 3–6 and appeared more thermally robust than the GH26 endomannanases with Tm values ranging from 78 to 87 °C and half-lives above 137 h. For the truncated enzymes, the thermal stability did not seem to be drastically influenced by the lack of the CBM, neither the CBM1 (*Tres*Man5A) or CBM35 (*Pans*Man26A) (Table 1). When compared at 37 °C, all the endomannanases had 79–100% relative activity at pH 5 compared to the activity at their pH optimum.

### Initial rates of enzymatic hydrolysis on pure mannans

**Fig. 2:**
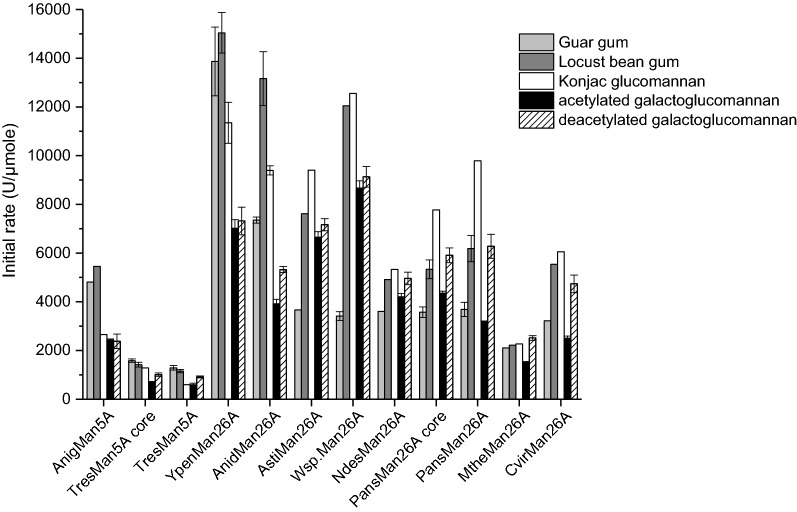
Initial reaction rates (U/µmole) by endomannanases on pure mannans (37 °C and pH 5). Guar gum (light grey), locust bean gum (dark grey), konjac glucomannan (white), acetylated spruce galactoglucomannan (black) and deacetylated spruce galactoglucomannan (lined). Values are given as mean values ± SD (*n* = 1–7). One-way ANOVA analyses can be seen in Additional file 1: Tables S1 and S2

The initial rates of endomannanase catalysed hydrolysis of locust bean gum, guar gum, konjac glucomannan, as well as acetylated and deacetylated spruce galactoglucomannan were determined at pH 5 and at 37 °C, to assess the activity without confounding effects of differential thermostability of the enzymes (Fig. 2). No *β*-glucanase cross-activity was found for any of the tested endomannanases, *Cvir*Man26A, *Pans*Man26A, *Pans*Man26A core, *Anig*Man5A, *Tres*Man5A and *Tres*Man5A core (assessed on barley *β*-glucan and carboxymethyl cellulose).

The endomannanases showed different activity levels on the pure mannan substrates.

GH5 endomannanases tended to exhibit lower initial rates than the GH26, but no consistent discrimination between the enzymes’ substrate preferences was evident (Fig. 2). *Ypen*Man26A had a significantly higher initial rate than all the other enzymes on the two galactomannans: locust bean gum (15,050 U/µmole) and guar gum (13,850 U/µmole). In contrast, *Wsp*Man26A had the highest initial rates of all tested endomannanases on the glucomannans: konjac glucomannan (12,550 U/µmole), acetylated galactoglucomannan (8650 U/µmole), and deacetylated galactoglucomannan (9150 U/µmole) (Fig. 2). Deacetylation of galactoglucomannan doubled the rate for a few GH26 endomannanases (*Pans*Man26A and *Cvir*Man26A), but did not generally affect rates or caused rate increase. The lowest initial rates were observed for *Tres*Man5A on the acetylated spruce galactoglucomannan and on konjac glucomannan (on both substrates 600 U/µmole). In general, *Tres*Man5A had lowest initial rates of all the endomannanases on all the tested mannans, irrespective of the presence of the CBM1. The lack of significance of the CBM1 presence is in accordance with *Tres*Man5A CBM1 being known to bind to cellulose and not to mannan [27]. In contrast, the initial rates of *Pans*Man26A containing a CBM35 tended to be higher than those for the *Pans*Man26A core, especially on locust bean gum and konjac glucomannan. A positive effect of the CBM35 may be related to the reported interaction of CBM35 with *β*-mannans or *α*-d-galactopyranosyl residues [30, 31]. It seems more likely that the *Pans*Man26A CBM35 interacts with the *β*-mannan backbone than with the *α*-d-galactopyranosyl substitutions, since the positive effect of the CBM was not found on the highly substituted guar gum.

### Differences in substrate preferences

The wild type core GH26 endomannanases, *Ypen*Man26A and *Anid*Man26A, had a significantly higher initial rate on locust bean gum (15,050 and 13,150 U/µmole) compared to on konjac glucomannan (11,350 and 9400 U/µmole). All other tested GH26 endomannanases had higher (or similar) initial rates on konjac glucomannan than on locust bean gum, including *Pans*Man26A core for which the CBM35 were removed artificially. *Ypen*Man26A and *Anid*Man26A do not have a CBM, hence the substrate preferences exhibited by these enzymes as compared to those with a CBM35 must be tied to the enzyme core properties rather than to presence of the CBM35 domain. Interestingly, Katsimpouras et al. [29] reported that the GH26 endomannanase with a CBM35 from *Myceliophthora thermophila* had similar substrate preference trends, i.e., showing higher activity on konjac glucomannan compared to locust bean gum.

Like the wild type core GH26 endomannanases, *Tres*Man5A and *Anig*Man5A, had significantly higher initial rates on locust bean gum (1150 and 5450 U/µmole) compared to on konjac glucomannan (600 and 2650 U/µmole). The data for the GH5 enzymes correspond with the substrate preferences reported for *A. nidulans* GH5 endomannanases [37].

### Endomannanase performance in softwood saccharification

**Fig. 3:**
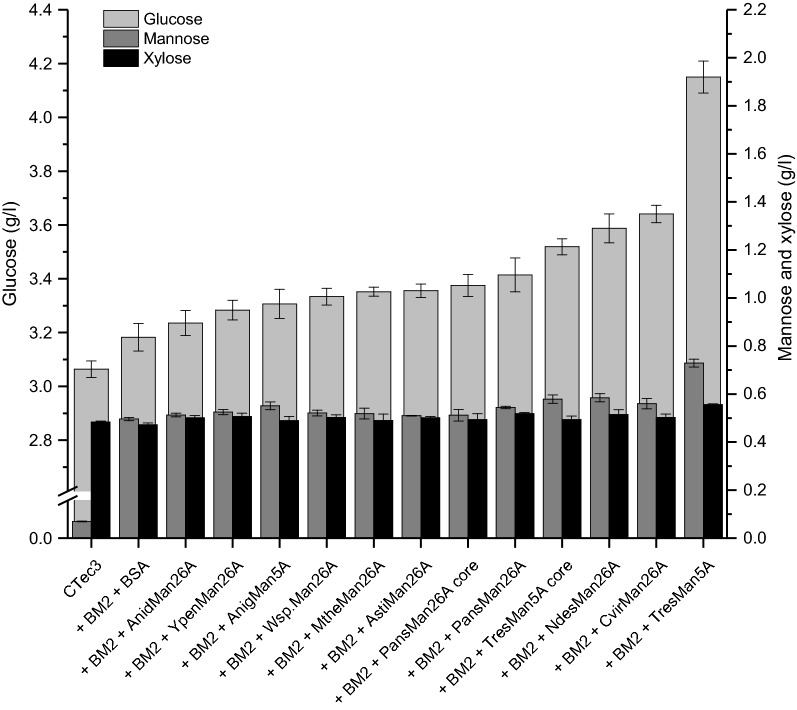
Softwood saccharification. Endomannanases or BSA were added in equal molar amounts on top of Cellic ^®^ CTec3 plus an *A. niger* GH2 *β*-mannosidase (BM2). Samples were taken after 24 h saccharification at 30 °C. Glucose (g/l, light grey), mannose (dark grey) and xylose (black) yields are given as mean values ± SD (*n* = 3). One-way ANOVA analyses can be seen in Additional file 1: Table S3. Yields from 48 and 144 h can be seen in Additional file 1: Figure S2

**Fig. 4:**
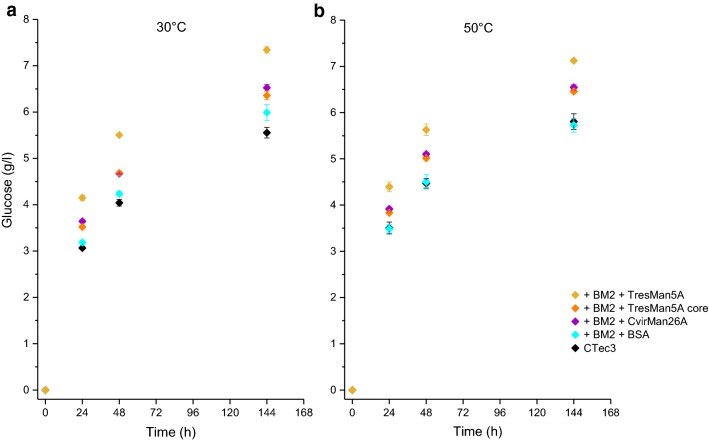
Softwood saccharification at 30 °C (**a**) and 50 °C (**b**) with *Tres*Man5A, *Tres*Man5A core, *Cvir*Man26A or BSA. Endomannanases or BSA were added in equal molar amounts on top of Cellic ^®^ CTec3 plus an *A. niger* GH2 *β*-mannosidase (BM2). Glucose yields (g/l) are given as mean values ± SD (*n* = 3). One-way ANOVA analyses can be seen in Additional file 1: Table S4

The efficiency of the 10 endomannanases for saccharification of softwood was assessed by adding equal molar amounts of each endomannanase on top of Cellic^®^ CTec3, where in each case the Cellic^®^ CTec3 had been supplied with a pure GH2 *β*-mannosidase from *Aspergillus niger* (BM2). When assessed on locust bean gum Cellic^®^ CTec3 itself exerted weak mannan-degrading activity. The endomannanase addition levels were ten times higher than this background activity. The release of glucose, mannose and xylose, respectively, was quantified at 24, 48 and 144 h (Figs. 3, 4 and Additional file 1: Figure S2). BSA was added as a protein control to assure that any differences in release of monosaccharides were not due to increased levels of protein as sometimes observed in lignocellulose hydrolysis (BSA binds non-productively) [38]. In a direct comparison of Cellic^®^ CTec3 with Cellic^®^ CTec3 plus BM2 plus BSA after 24 h hydrolysis, the *β*-mannosidase itself profoundly increased the release of mannose from 0.07 to 0.5 g/l (0.43 g/l increase) and the release of glucose from 3.06 to 3.18 g/l (0.12 g/l increase) (Fig. 3).

The increased mannose and glucose release is most likely due to BM2 activity on soluble galactoglucomannan oligosaccharides in the mixtures. By removing mannopyranosyl units from the nonreducing end of these oligosaccharides, the BM2 will expose glucopyranosyl residues in the nonreducing end, which can be released by *β*-glucosidase activity from the Cellic^®^ CTec3. The released amount of mannose and glucose upon addition of BM2 corresponds to a Man:Glc ratio of 3.6:1. This ratio is in agreement with reported Man:Glc ratios in softwood (spruce) galactoglucomannans [11], suggesting that no additional cellulose was degraded. The galactoglucomannan oligosaccharides were most likely released by low endomannanase activity present in the Cellic^®^ CTec3 preparation and/or by weak glucomannan degrading capacity by some endoglucanases of this enzyme cocktail (Fig. 1).

Supplementation of Cellic^®^ CTec3 with endomannanase significantly increased the release of glucose for all tested endomannanases, with *Tres*Man5A being the best performing candidate. After 24 h of enzyme treatment, the release of glucose and mannose obtained with the *Tres*Man5A addition was 30% (increase in 1 g/l glucose) and 15% (increase in 0.23 g/l mannose) higher than that of the control (Cellic^®^ CTec3 + BM2 + BSA, Fig. 3), and much higher than those obtained with any of the other endomannanases. The relative amount of released glucose and mannose (Man:Glc, 0.2:1) infer that the released glucose did not derive solely from hydrolysed galactoglucomannan, but also from the cellulose fraction. The increased cellulose degradation obtained with *Tres*Man5A after 24 h, was apparently accompanied by a slightly increased xylose release as well (Additional file 1: Table S3). As discussed below, the overall impression was that the mannose and xylose and the glucose and xylose release were linearly correlated.

The second-best enzyme was *Cvir*Man26A, containing both CBM35 and CBM1, with a glucose yield of 88% of that obtained by *Tres*Man5A (Fig. 3). The release of glucose and mannose continued throughout the 144 h hydrolysis (Additional file 1: Figure S2).

No obvious trends in the effect of GH5 versus GH26 endomannanases could be discerned. *Tres*Man5A was the superior enzyme, but glucose yields obtained with the other GH5 endomannanase, *Anig*Man5A, were in the low–middle range. For *Tres*Man5A, the presence of CBM1 improved the release of both mannose and glucose. However, both *Cvir*Man26A and *Asti*Man26 with a CBM1 caused release of medium levels of glucose, but there were no evident differences in their mannose release compared to the other endomannanases. No significant effect of the presence of CBM35 was observed for *Pans*Man26A.

Enzyme robustness does not explain the observed difference between the boosting capacity of the investigated endomannanases (Table 1 and Fig. 3). For example, *Tres*Man5A and *Cvir*Man26A had the same stable nature during 48 h incubation at 30 °C (Table 1 and Additional file 1: Figure S1), but differed in their boosting capacity. However, enzyme robustness possibly contributed to some of the observed differences in the enzymes’ boosting capacities. For example, the low stability of *Ypen*Man26A and *Anid*Man26A (Table 1 and Additional file 1: Figure S1) may partially explain their poor overall performance in boosting of glucose release from softwood (Fig. 3).

### Saccharification at 50 °C

When the two endomannanases, *Tres*Man5A and *Cvir*Man26A, were assessed at 50 °C, it was confirmed that they both boosted the glucose release catalysed by Cellic^®^ CTec3 and that the complete *Tres*Man5A with the CBM1 induced release of significantly more glucose than the *Cvir*Man26A and the *Tres*Man5A core. The time curves of the enzymatic glucose release at 50 and 30 °C were in complete agreement (Fig. 4), and the ranking of the performance of the enzymes was similar at the two reaction temperatures. The lack of increase in hydrolytic rate by cellulases in Cellic^®^ CTec3 with temperature (Q_10_ close to 1 between 30 and 50 °C) is in agreement with data published by Westh et al. [39, 40] who showed that at low Avicel concentrations reduction in substrate affinity caused by heating (increase in *K*_M_) cancels thermoactivation (increase in *k*_cat_). In the present study, the substrate concentration was low (2% DM ~ 1% cellulose). The effective accessible substrate concentration may have been lower, because not all cellulose is equally accessible. Since industrial lignocellulose conversion is usually executed at 50 °C, the data strongly indicate that addition of *Tres*Man5A to commercial cellulase preparations can efficiently boost glucose yields in industrial softwood saccharification reactions.

### Correlation between release of glucose, mannose and xylose

With the Cellic ^®^ CTec3 and endomannanase doses used (Table 3), the maximal degree of conversion was approximately 60% of glucose (7.3 g/l of the available 11.5 g/l of glucose were released) after 144 h (Additional file 1: Figure S2). To assess the softwood saccharification at a higher degree of conversion, the enzyme loadings were increased, i.e., addition levels of *Tres*Man5A and *Cvir*Man26A, respectively, and the Cellic ^®^ CTec3 dose were increased (Table 3). With the high enzyme doses, 85% cellulose, 60% mannan, and 81% xylan conversion were obtained at 30 °C after 144 h.

**Fig. 5:**
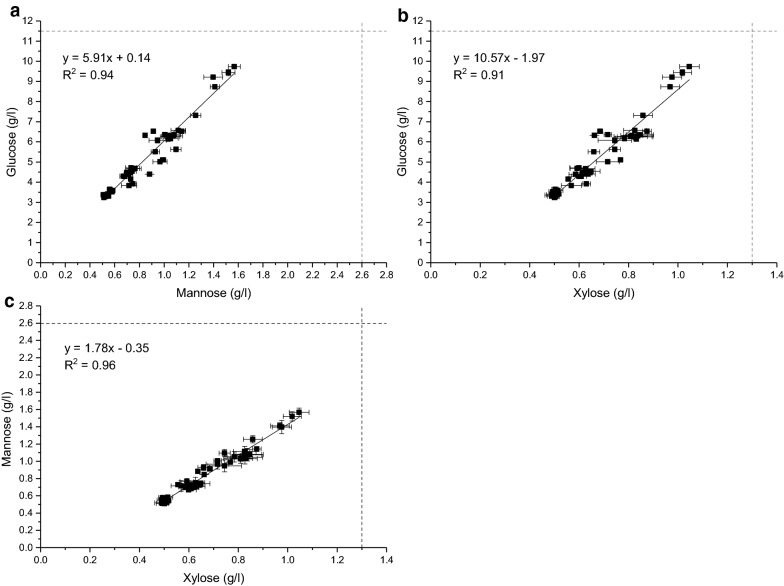
Correlation between the release (g/l) of glucose and mannose (**a**), glucose and xylose (**b**), and mannose and xylose (**c**) during softwood saccharification. Dashed lines show the theoretical monomeric yield of the substrate based on the chemical composition analysis (Table 2)

The data obtained, plus the saccharification results presented in Figs. 3, 4, and Additional file 1: Figure S2, showed a clear linear correlation between the release of glucose, mannose, and xylose throughout the degradation (Fig. 5). These results support the comprehension that the softwood substrate comprises a complex network with glucomannans and xylans located throughout the lignocellulose matrix [3], and not on the outer surface. Their concurrent hydrolysis is crucial to obtain extensive hydrolysis of cellulose and in turn maximize the overall glucose yields from the softwood substrate. A reason for the lower conversion of mannan (approximately 60%) than glucan and xylan (approximately 80%) might be related to the galactose substitutions on galactoglucomannans that hinder the *β*-mannosidase in fully degrading the released mannooligosaccharides to mannose (Fig. 1), which in turn would explain why not all solubilized galactoglucomannan was analysed as monomers.

### Performance in softwood saccharification was not predicted by initial rate

When comparing the initial rates on the soluble model substrates and the boosting effect, the general trend is that the GH5 enzymes show low initial rates but in the case of *Tres*Man5A comparably high boosting effect. For the GH26 enzymes the situation appears more complex, although high initial rate (e.g., *Ypen*Man26A) is not correlated to high boosting effect. Despite its high boosting capacity, *Tres*Man5A was found to have the lowest initial rate on the pure mannans (Fig. 2), demonstrating that performance comparison on these substrates could not predict the efficiency of the enzymes in softwood saccharification. Earlier studies have proposed differences in the biological role for bacterial GH5 and GH26 endomannanases, with the GH5 being optimal for degradation of cell wall mannans [22, 23]. The present study suggests that the GH family categorization and the biological role is not as clear cut for the fungal endomannanases. It was a GH26 endomannanase that performed best on the pure and soluble mannans and a GH5 on the cell wall substrate. However, most of the tested GH26 endomannanases performed on par with, or even better than the *Anig*Man5A. The fact that some fungal GH26 endomannanases are found with a CBM1 and that we have seen no sign of cell wall association for these enzymes, also indicate that the fungal GH26 endomannanases participate in cell wall degradation in nature.

In general, the hydrolysis rate of mannan during softwood saccharification was much lower than the initial hydrolysis rates measured on the pure mannans. During the first 24 h, the mannose release from the softwood substrate by *Tres*Man5A and BM2 reached 0.73 g/l (Fig. 3). If averaging over 24 h, this amount corresponds to a hydrolysis rate on 2.8 U/l (approximately 11 U/µmole *Tres*Man5A). This is likely an overestimated rate for the endomannanase since it attacks the mannan backbone, while it is the surplus of BM2 that causes the release of mannose. On the other hand, the rate is probably not constant throughout the first 24 h, but higher during the initial reaction period. Even if the initial reaction rate for *Tres*Man5A on the softwood substrate was 20 U/µmole, this rate is still 45 times lower than the initial rates obtained for *Tres*Man5A on extracted deacetylated spruce galactoglucomannan at 900 U/µmole (the acetyl moieties are expected to be lost during pretreatment of the softwood). On this insoluble lignocellulosic matrix, the hydrolysis rate of the endomannanases is comparable with the rate of cellulases working on insoluble cellulose [39]. Neither the initial rate itself nor particular substrate preferences of the individually endomannanases with regard to galactose substitutions or acetylation, seemed to determine their performance in softwood saccharification. This in turn means that other properties of the enzymes must be considered.

### Explaining the high boosting effect of *Tres*Man5A in softwood saccharification

Our results clearly show that *Tres*Man5A with its CBM1 was the most efficient for softwood saccharification among the tested endomannanases (when added to Cellic ^®^ CTec3). It has previously been observed that hemicellulases from *T. reesei* reduce hemicellulose exposed at the cellulose surface of wood materials to a greater extent than hemicellulases from *Aspergillus* sp. [41], and that *Tres*Man5A catalyses hydrolysis of softwood galactoglucomannan [17]. Our hypothesis for a mechanistic explanation about the additional boosting effect is that *Tres*Man5A catalyses a faster or more profound degradation of a certain type of mannan that is not immediately accessible for the other endomannanases and which moreover, when degraded, allows for a more profound cellulose degradation. Since at least part of softwood mannan is closely associated with cellulose [42], it cannot be ruled out that this is the case also with the pretreated material. The particular portion of the mannan may be a more crystalline part that is more tightly intertwined with the cellulose. It is likely, that the CBM1 in the full-length *Tres*Man5A helps target the cellulose-associated mannan more efficiently than its truncated counterpart lacking CBM1. This view is supported by a previous study of *Tres*Man5A action on cellulose-mannan complexes and the CBM1 cellulose binding capacity [27]. When plant cell wall material are enzymatically degraded by endomannanases and other glycoside hydrolases, CBMs are in general important for ensuring correct positioning and close proximity between enzymes and glycan substrates in turn facilitating enzymatic hydrolysis by the catalytic core modules [22].

Since the *Tres*Man5A core without the CBM1 was among the top performers in the softwood saccharification, the core module itself also played a role for the efficient degradation of the insoluble mannans. The reason that the other GH26 endomannanases with a CBM1, i.e., *Cvir*Man26A and *Ast*Man26, were not releasing the same levels of mannose and glucose as *Tres*Man5A, could be because their core modules are not as optimal as *Tres*Man5A for degradation of mannan associated to cellulose. Another reason might be that their CBM1s have slightly different specificities than the *Tres*Man5A CBM1.

## Conclusion

This study strongly confirms that fungal endomannanases differ in their capacity to degrade galactoglucomannan in softwood, and that this degradation contributes significantly to obtain increased enzymatic cellulose saccharification of softwood. Apart from this main finding, the key novel result was that the well-studied *Tres*Man5A was superior to all other tested GH5 and GH26 endomannanses (also with CBM1 modules) in genuine softwood saccharification, despite being among the slowest on purified mannan substrates. Two GH5 and eight GH26 endomannanases (including six novel endomannanases) were successfully recombinantly expressed, purified, and characterized with focus on their performance in softwood saccharification. The fungal GH26 endomannanases from *Yunnania penicillata* and *Westerdykella* sp. were found to have highest initial rates among the tested enzymes on pure soluble galactomannans and glucomannans, respectively. The acetyl groups on extracted spruce galactoglucomannan tended to decrease the initial enzymatic rates when compared to the initial rates on the deacetylated substrate. However, these initial rates on the pure mannans did not correlate with the results obtained in the extended softwood saccharification reactions. All tested endomannanases caused increased glucose release during softwood saccharification when compared to the glucose release catalysed by Cellic ^®^ CTec3 plus the *A. niger β*-mannosidase alone. However, the GH5 endomannanase from *T. reesei* with a CBM1 produced a markedly higher mannose and glucose release at all time points than all the other tested endomannanases. Based on the data, our hypothesis is that *Tres*Man5A is able to attack an additional portion of mannan in the lignocellulosic matrix, allowing for better cellulose degradation. Both the catalytic efficiency of the core module and the presence of the CBM1 play important roles in the superior performance of this enzyme on softwood. The other nine GH5 and GH26 endomannanases performed on par with the softwood lignocellulosic matrix, giving no clear signs of different biological roles for these fungal endomannanases. Presence of the CBM35 did not change the performance of *Pans*Man26 in softwood saccharification.

The data obtained highlight the problematic strategy of selecting enzymes for industrial applications based on basic characterisation on pure and well-defined substrates. Neither rates nor substrate preferences observed in the basic characterisation correlated with efficient softwood saccharification. Evidently, the data of this study have implications for the selection and use of endomannanases in industrial softwood saccharification applications, especially as new pretreatment methods leaves more hemicellulose in the lignocellulosic matrix after pretreatment.

## Methods

### Materials

Locust bean gum (low viscosity; borohydride reduced), guar gum (high viscosity), konjac glucomannan (high viscosity), *β*-glucan (barley; high viscosity), and carboxymethyl cellulose, were purchased from Megazyme (Ireland). Spruce galactoglucomannan (Man:Glc:Gal:Ac, 3.3:1:0.83:1.32) was prepared as described previously [43]. The *O*-acetyl moieties of the *O*-acetylated spruce galactoglucomannan were removed by alkaline hydrolysis in the presence of ammonium hydroxide as described by Jacobs et al. [44]. All other chemicals were from Sigma (Germany).

### Expression and purification

The fungal GH26 endomannanases from *Collariella virescens* (*Cvir*Man26A), *Mycothermus thermophiles* (*Mthe*Man26A), *Neoascochyta desmazieri* (*Ndes*Man26A), *Ascobolus stictoideus* (*Asti*Man26A), *Westerdykella* sp. (*Wsp.*Man26A), *Aspergillus nidulans* (*Anid*Man26A), *Yunnania penicillata* (*Ypen*Man26A), *Podospora anserina* (*Pans*Man26A and *Pans*Man26A core), the fungal GH5 endomannanases from *Aspergillus niger* (*Anig*Man5A), and *Trichoderma reesei* (*Tres*Man5A and *Tres*Man5A core) were recombinantly expressed in *Aspergillus oryzae* MT3568an amdS [45]. *Pans*Man26 core and *Tres*Man5A core were expressed without the linker and the N-terminal CBM35 and the C-terminal CBM1, respectively. The enzymes were purified to electrophoretic purity using hydrophobic interaction and ion exchange chromatography (SDS-PAGE gels shown in Additional file 1: Figure S3). The identity of the purified endomannanases was validated with mass spectrometry analysing a tryptic digest of the protein band excised from a SDS-PAGE gel. Protein concentrations were determined by UV absorption at 280 nm using theoretical extinction coefficients (*ε*). *ε* at 280 nm of all proteins were estimated by GPMAW 9.20 (Lighthouse Data), and were based on mature proteins without modifications.

### pH optimum

The hydrolytic activity was determined at 37 °C, after 15 min, over a pH range from 2.0 to 12.0, with 1 pH unit intervals. The hydrolysis volume was 200 µl, with 2.5 mg/ml locust bean gum in a buffer with 50 mM acetic acid, 50 mM HEPES, 50 mM glycine, 0.01% Trition X-100, 50 mM potassium chloride and 1 mM calcium chloride. The buffer pH was adjusted with sodium hydroxide from pH 2.0–12.0. Released reducing sugars were measured with the 4-hydroxybenzoic acid hydrazide (PAHBAH) method described by Lever [46], with mannose as standard.

### Initial rates

The initial rate on locust bean gum, guar gum, konjac glucomannan, acetylated and deacetylated spruce galactoglucomannan, *β*-glucan, and carboxymethyl cellulose by the endomannanases were determined with 2.5 mg/ml substrate in 50 mM sodium acetate pH 5 at 37 °C. All substrates, except the deacetylated spruce galactoglucomannan and the carboxymethyl cellulose, were soluble at the concentration employed. The deacetylated spruce galactoglucomannan and the carboxymethyl cellulose were only partly soluble, but these substrates were kept in suspension during reaction and sampling via vigorous mixing. Released reducing sugars were measured with the PAHBAH method as described above, except that glucose was used as standard for measurements on *β*-glucan and carboxymethyl cellulose. All hydrolysis assays were carried out at seven different endomannanase doses as described elsewhere [26]. Initial rates were calculated in the initial linear range of the hydrolysis. To validate that the slope calculation was reproducible, up to seven replicates were done for selected enzymes on selected substrates. The CV was below 10%. The initial rate by the other enzymes was calculated from one slope. One unit (U) was defined as the amount of endomannanase required to release 1 µmole of reducing ends per minute, under the assay conditions specified.

### Thermal stability

The thermal stability at pH 5 was investigated with differential scanning calorimetry (DSC) as described elsewhere [26]. The thermal midpoint (Tm) was determined as the top of the protein denaturation peak, and was determined at an accuracy of ± 1 °C. To assay enzyme stability at 30 °C, which was the temperature used in the softwood saccharification reactions, the purified enzymes were incubated individually in triplicates at 30 °C, in 50 mM sodium acetate pH 5, for 24 h and 48 h. Residual activity was determined at 30 °C, pH 5, on locust bean gum with the assay conditions described above.

### Biomass and pretreatment

Commercially available grey-stage beetle killed lodgepole pinewood (LPP) chips (*Pinus contorta*) were pretreated, and the chemical composition was analyzed (pretreatment and compositional analysis was done by University of British Colombia, Vancouver, Canada). Wood chips were screened and a size fraction between 2.5 × 2.5 and 5.0 × 5.0 cm was collected and used as feedstock for pretreatment. The pretreatment was performed in a similar manner to the procedure developed by Chandra et al. [35] which was shown to preserve the hemicellulose component in the water insoluble substrate. Prior to steam pretreatment, 200 g of LPP chips (8% moisture) were placed in thermal plastic bags, and mixed with 200 ml of water containing 6% sodium sulfite and 4% sodium carbonate (w/w based on dry wood). The bag of chips was sealed and submerged in a water bath at 60 °C for 12 h. The wet chemical impregnated biomass was then loaded to a 2-l Stake Tech II steam gun (Stake Tech II batch reactor, SunOpta of Norval, ON, Canada) and pretreated at 130 °C for 30 min. After steaming, the biomass remained as chips which were filtered, suspended in 20 l of water and then subjected to mechanical size reduction using a commercial juicer (Angel model 8500). After this process, the sample was filtered with the water insoluble fraction subsequently characterized for its chemical composition (Table 2) by the NREL method [47] and then used for enzymatic hydrolysis experiments.

**Table 2:** Chemical composition of the softwood dry matter (DM)

Softwood component	Percent of DM (%)
Arabinose	1.3
Galactose	1.3
Glucose	52
Xylose	5.5
Mannose	11.7
Lignin	27

### Enzymes for softwood hydrolysis

The applied enzymes were the purified endomannanases (see section about “Expression and purification”), a purified *A. niger* GH2 *β*-mannosidase (BM2, UNIPROT A2QWU9), and a commercially available cellulase- and xylanase-rich enzyme cocktail (Cellic^®^ CTec3). Except for the endomannanases, which were purified in this study, the enzymes were kindly provided by Novozymes. The applied enzyme doses can be seen in Table 3. Cellic^®^ CTec3 is a *Trichoderma*-based product with different recombinant enzymes. One can assume that the mannan-degrading activity from Cellic^®^ CTec3 is equivalent to *Tres*Man5A. Cellic^®^ CTec3 supplies approximately 1.3 × 10 ^−9^ mol/g dry matter (DM) endomannanase (*Tres*Man5A) to the softwood hydrolysis, when added in a concentration of 10 mg Cellic^®^ CTec3/g DM. The lowest addition of purified endomannanase was 1.26 × 10^−8^ mol/g DM.

**Table 3:** Doses of enzymes, enzyme cocktail and BSA in softwood saccharification

Set-up^a^	CTec3^b^	BM2	Endomannanase or BSA
	mg EP/g DM	mg EP/g DM	mol/g DM
(1)	10	1	1.26 × 10^−8^
(2)	50	1	1.26 × 10^−7^

^a^Two set-ups were used: comparing endomannanases at 30 and 50 °C (1). Increased enzyme doses to evaluate saccharification at a higher degree of conversion (2)

^b^Cellic^®^ CTec3 and the *A. niger* GH2 *β*-mannosidase (BM2) doses are given as mg enzyme protein (EP)/g dry matter (DM) and not as mg product

### Enzymatic hydrolysis of pretreated softwood

Enzymatic hydrolysis of the pretreated softwood was performed in 50 ml falcon tubes with two metal balls (9 mm). Softwood was added to give 0.4 g dry matter per tube (resulting in 2% dry matter) along with sodium acetate buffer, pH 5, and proxel to give concentrations of 50 mM and 0.2%, respectively, in the final mixture. Milli-Q water was added to give a total reaction mass of 20 g after addition of the required amount of enzyme. The tubes were incubated in a heated (30 or 50 °C) 20 cm diameter drum, rotating at 20 rpm. All experiments were performed in triplicates and run for 144 h. At sampling, 2 ml representative whole slurry sample was transferred to Eppendorf tubes and centrifuged 18,213*g* for 10 min. The liquid was decanted, filtered through a 0.45 and a 0.22 µm filter and kept for sugar analysis on high-performance liquid chromatography (HPLC) for separation and quantification of glucose amount and by 1-phenyl-3-methyl-5-pyrazolone (PMP) derivatization followed by reverse phase ultra-performance liquid chromatography (UPLC) for separation and quantification of mannose and xylose amounts.

### Analysis of sugar release

The amount of glucose was analysed by a Waters HPLC system coupled with a refractive index detector and equipped with a Aminex HPX 87H column (300 by 7.8 mm). 10 µl sample was injected and separation was performed at 65 °C with a flow rate of 0.6 ml/min 5 mM H_2_SO_4_. Calibration curves for glucose were plotted (Empower) and used to estimate the amount of glucose released.

The derivatization reaction, for analysing the amount of mannose and xylose, was performed with 200 µl sample in an appropriate concentration, 20 µl 6-deoxy-d-glucose as internal standard, 20 µl 4 M NaOH, and 200 µl 0.5 M PMP in aqueous methanol. The mixtures were mixed well and incubated at 70 °C for 30 min, cooled to room temperature and mixed with 20 µl 4 M HCL and 400 µl methanol. The separation and quantification of mannose and xylose was analysed by a Waters Acquity UPLC system coupled with a UV (245 nm) detector and equipped with a Waters Acquity CSH C18 column (dimensions: 150 × 2.1 mm, particle size: 1.7 µm and pore size: 130 Å). 3 µl sample was injected and separation was performed at 65 °C with a flow rate of 0.5 ml/min. A two-eluent system was used, (A) 0.15% formic acid in MiliQ water and (B) 0.15% formic acid in ACN with the following gradient: 0 min, 83:17 (% A:B); 1 min, 83:17 (% A:B); 10 min, 77.2:22.8 (% A:B); 10.5 min, 5:95 (% A:B); 11 min, 83:17 (% A:B). The total run time per injection was 13 min. Calibration curves for mannose and xylose were plotted (Empower3) and used to estimate the amount of released mannose and xylose. All results are expressed in g/l.

## Additional file

**Additional file 1: Table S1 and S2.** Ratings from one-way ANOVA analyses of initial reaction rates by endomannanases on pure mannans (Fig. 2). A one-way ANOVA analysis was made for all endomannanases on each substrate (top, Table S1) and for each enzyme across all substrates (bottom, Table S2). Ratings are assigned with a 95 % confidence interval with the Tukey–Kramer method in SASjmp. Please consult the manuscript for enzyme abbreviations. **Table S3.** Ratings from one-way ANOVA analyses of sugar release during softwood saccharification at 30 °C (Fig. 3 and Additional file 1: Figure S2). A one-way ANOVA analysis was made for each sugar at each time point. Ratings are assigned with a 95 % confidence interval with the Tukey–Kramer method in SASjmp. Please consult the manuscript for enzyme abbreviations. **Table S4.** Ratings from one-way ANOVA analyses of glucose release during softwood saccharification at 30 and 50 °C (Fig. 4). A one-way ANOVA analysis was made for each temperature at each time point. Ratings are assigned with a 95 % confidence interval with the Tukey–Kramer method in SASjmp. Please consult the manuscript for enzyme abbreviations. **Figure S1.** Stability of endomannanases at 30 °C. The relative activity (%) was determined on locust bean gum at 30 °C, pH 5 and are given as mean values ± SD (n=3). Please consult the manuscript for enzyme abbreviations. **Figure S2.** Softwood saccharification. Endomannanases or BSA were added in equal molar amounts on top of Cellic ^®^ CTec3 plus an *A. niger* GH2 *β*-mannosidase (BM2). Samples were taken after 24, 48 and 144 h saccharification at 30 °C. Glucose (g/l, light grey), mannose (dark grey) and xylose (black) yields are given as mean values ± SD (n=3). One-way ANOVA analyses can be seen in Additional file 1: Table S3. **Figure S3.** SDS-PAGE gels of the purified enzymes. The protein concentration in the samples was 0.5 mg/ml. Prior to gel loading, samples were diluted 1:1 with loading mix. Loading mix was prepared as a 9:1 mix of Novex ® Tris-Glycine SDS Sample Buffer (2X) (Life Technologies) and Nupage ® Sample Reducing Agent (10X) (Life Technologies). Please consult the manuscript for enzyme abbreviations. Samples with *Ndes*Man26A and *Mthe*Man26A both contain molecules with and without the CBM35.
