# Supplementary material for: Boosting of enzymatic softwood saccharification by fungal GH5 and GH26 endomannanases
Source: Biotechnol Biofuels. 2018 Jul 17;11:194. doi: 10.1186/s13068-018-1184-y (PMC6048861; doi:10.1186/s13068-018-1184-y)
Supplement: Supplementary file 1 — Additional file 1: Table S1 and S2. Ratings from one-way ANOVA analyses of initial reaction rates by endomannanases on pure mannans (Fig. 2). A one-way ANOVA analysis was made for all endomannanases on each substrate (top, Table S1) and for each enzyme across all substrates (bottom, Table S2). Ratings are assigned with a 95 % confidence interval with the Tukey–Kramer method in SASjmp. Please consult the manuscript for enzyme abbreviations. Table S3. Ratings from one-way ANOVA analyses of sugar release during softwood saccharification at 30 °C (Fig. 3 and Additional file 1: Figure S2). A one-way ANOVA analysis was made for each sugar at each time point. Ratings are assigned with a 95 % confidence interval with the Tukey–Kramer method in SASjmp. Please consult the manuscript for enzyme abbreviations. Table S4. Ratings from one-way ANOVA analyses of glucose release during softwood saccharification at 30 and 50 °C (Fig. 4). A one-way ANOVA analysis was made for each temperature at each time point. Ratings are assigned with a 95 % confidence interval with the Tukey–Kramer method in SASjmp. Please consult the manuscript for enzyme abbreviations. Figure S1. Stability of endomannanases at 30 °C. The relative activity (%) was determined on locust bean gum at 30 °C, pH 5 and are given as mean values ± SD (n=3). Please consult the manuscript for enzyme abbreviations. Figure S2. Softwood saccharification. Endomannanases or BSA were added in equal molar amounts on top of Cellic ® CTec3 plus an A. niger GH2 β-mannosidase (BM2). Samples were taken after 24, 48 and 144 h saccharification at 30 °C. Glucose (g/l, light grey), mannose (dark grey) and xylose (black) yields are given as mean values ± SD (n=3). One-way ANOVA analyses can be seen in Additional file 1: Table S3. Figure S3. SDS-PAGE gels of the purified enzymes. The protein concentration in the samples was 0.5 mg/ml. Prior to gel loading, samples were diluted 1:1 with loading mix. Loading mix was prepared as a 9:1 mix of No [file 13068_2018_1184_MOESM1_ESM.docx]

**Additional material**

**Boosting of enzymatic softwood saccharification by** fungal GH5 and GH26 **endomannanases**

von Freiesleben et al.

**Table S1 and S2** Ratings from one-way ANOVA analyses of initial reaction rates by endomannanases on pure mannans (Figure 2). A one-way ANOVA analysis was made for all endomannanases on each substrate (top, Table S1) and for each enzyme across all substrates (bottom, Table S2). Ratings are assigned with a 95 % confidence interval with the Turkey-Kramer method in SASjmp. Please consult the manuscript for enzyme abbreviations.

**Table S1**

| **Endomannanases** | **Guar gum** | **Locust bean gum** | **Konjac glucomannan** | **Acetylated GGM ^a^** | **Deacetylated GGM ^a^** |
| --- | --- | --- | --- | --- | --- |
| *Ypen*Man26A | A | A | A | B | B |
| *Wsp.*Man26A | C, D | B | A | A | A |
| *Anid*Man26A | B | B | A, B | C | D, E |
| *Asti*Man26A | C, D | C | A, B, C, D | B | B, C |
| PansMan26A | C | C | A, B, C | C, D | B, C, D |
| *Pans*Man26A core | C, D | C | A, B, C, D | C | C, D, E |
| *Ndes*Man26A | C, D | C, D | A, B, C, D | C | E |
| *CvirMan26A* | C, D | C | A, B, C, D | D, E | E |
| *Anig*Man5A | C | C | B, C, D | D, E | F |
| *Mthe*Man26A | D, E | D, E | B, C, D | E, F | F |
| *Tres*Man5A core | E | E | C, D | F | G |
| *TresMan5A* | E | E | D | F | G |

**Table S2**

| **Endomannanases** | **Guar gum** | **Locust bean gum** | **Konjac glucomannan** | **Acetylated GGM ^a^** | **Deacetylated GGM ^a^** |
| --- | --- | --- | --- | --- | --- |
| *Ypen*Man26A | A, B | A | B | C | C |
| *Wsp.*Man26A | C | A | A | B | B |
| *Anid*Man26A | B, C | A | B | D | C, D |
| *Asti*Man26A | C | A, B | A | B | A, B |
| PansMan26A | C | B | A | C | B |
| *Pans*Man26A core | C | B | A | B, C | B |
| *Ndes*Man26A | A | A | A | A | A |
| *CvirMan26A* | A, B | A | A | B | A |
| *Anig*Man5A | A, B | A | B, C | C | C |
| *Mthe*Man26A | A, B | A | A | B | A |
| *Tres*Man5A core | A | A, B | B, C | D | C |
| *TresMan5A* | A | A | B, C | C | B |

^a^ spruce galactoglucomannan (GGM)

**Table S3** Ratings from one-way ANOVA analyses of sugar release during softwood saccharification at 30 °C (Figure 3 and Figure S2). A one-way ANOVA analysis was made for each sugar at each time point. Ratings are assigned with a 95 % confidence interval with the Turkey-Kramer method in SASjmp. Please consult the manuscript for enzyme abbreviations.

| **Samples** | **24h** | | | **48h** | | | **144h** | | |
| --- | --- | --- | --- | --- | --- | --- | --- | --- | --- |
|  | **Glucose** | **Mannose** | **Xylose** | **Glucose** | **Mannose** | **Xylose** | **Glucose** | **Mannose** | **Xylose** |
| *+ BM2 + Tres*Man5A | A | A | A | A | A | A | A | A | A |
| *+ BM2 + Cvir*Man26A | B | B, C | B, C | B, C | B | A, B | B, C | A, B | A |
| *+ BM2 + Ndes*Man26A | B | B | A, B | B | B | A, B | B | A, B, C | A, B |
| *+ BM2 + Tres*Man5A core | B, C | B | B, C | B | B | A, B | B, C, D | B, C, D | B |
| *+ BM2 +* PansMan26A | C, D | B, C, D | A, B | B, C, D | B | A | B, C, D, E | B, C, D | A, B |
| *+ BM2 + Pans*Man26A core | D | D, E | B, C | C, D | B | A, B | B, C, D, E | B, C, D | A, B |
| *+ BM2 + Asti*Man26A | D, E | D, E | B, C | D, E | B | A, B | B, C, D | B, C, D | A, B |
| *+ BM2 + Mthe*Man26A | D, E | C, D, E | B, C | D, E, F | B | A, B | B, C, D, E | B, C, D | A, B |
| *+ BM2 + Wsp.*Man26A | D, E | C, D, E | B, C | D, E, F, G | B | A, B | B, C, D, E | B, C, D | A, B |
| *+ BM2 + Anig*Man5A | D, E | B, C, D | B, C | D, E, F | B | A, B | C, D, E | B, C, D | A, B |
| *+ BM2 + Ypen*Man26A | D, E, F | C, D, E | B, C | E, F, G | B | A, B | C, D, E | B, C, D | A, B |
| *+ BM2 + Anid*Man26A | E, F | C, D, E | B, C | F, G | B | A, B | D, E | D | A, B |
| *+ BM2 + BSA* | F | E | C | G | B | A, B | E | C, D | A, B |
| *CTec3* | G | F | B, C | H | C | B | F | E | A, B |

**Table S4** Ratings from one-way ANOVA analyses of glucose release during softwood saccharification at 30 and 50 °C (Figure 4). A one-way ANOVA analysis was made for each temperature at each time point. Ratings are assigned with a 95 % confidence interval with the Turkey-Kramer method in SASjmp. Please consult the manuscript for enzyme abbreviations.

| **Samples** | **24 h** | | **48 h** | | **144 h** | |
| --- | --- | --- | --- | --- | --- | --- |
|  | **30 °C** | **50 °C** | **30 °C** | **50 °C** | **30 °C** | **50 °C** |
| *+ BM2 + TresMan5A* | A | A | A | A | A | A |
| *+ BM2 + CvirMan26A* | B | B | B | B | B | B |
| *+ BM2 + TresMan5A core* | B | B | B | B | B | B |
| *+ BM2 + BSA* | C | C | C | C | C | C |
| *CTec3* | D | C | D | C | D | C |


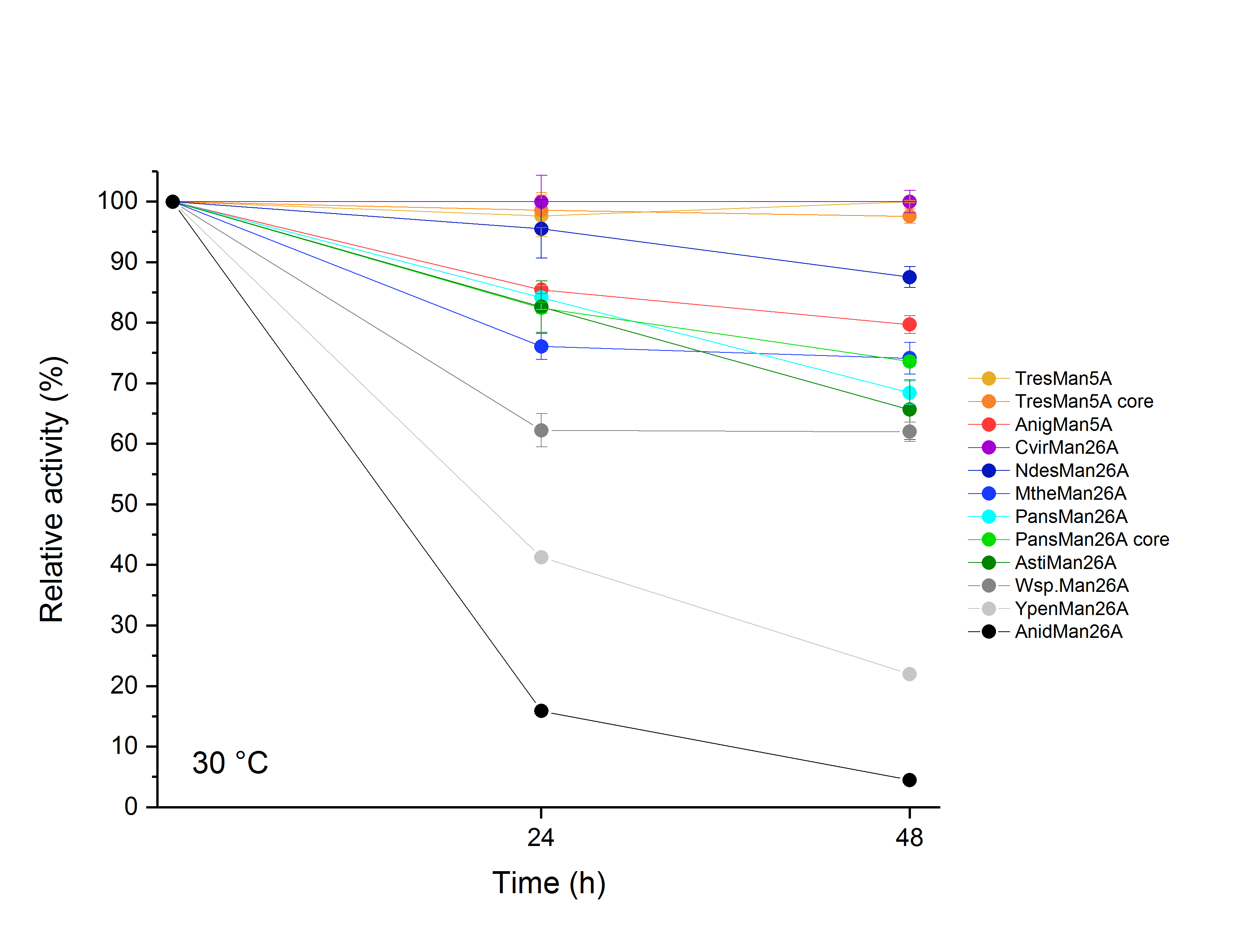


**Figure S1** Stability of endomannanases at 30 °C. The relative activity (%) was determined on locust bean gum at 30 °C, pH 5 and are given as mean values ± SD (n=3). Please consult the manuscript for enzyme abbreviations.





**Figure S2** Softwood saccharification. Endomannanases or BSA were added in equal molar amounts on top of Cellic ^®^ CTec3 plus an *A. niger* GH2 *β*-mannosidase (BM2). Samples were taken after 24, 48 and 144 h saccharification at 30 °C. Glucose (g/l, light grey), mannose (dark grey) and xylose (black) yields are given as mean values ± SD (n=3). One-way ANOVA analyses can be seen in Table S3.

| \| **Lane** \| **Sample** \| \| --- \| --- \| \| 1 \| *Anid*Man26A – 5µl \| \| 2 \| LMW – 10µl \| \| 3 \| *Pans*Man26A – 5µl \| \| 4 \| LMW – 10µl \| \| 5 \| *Pans*Man26A core – 5µl \| \| 6 \| LMW – 10µl \| \| 7 \| *Tres*Man5A – 5µl \| \| 8 \| LMW – 10µl \| \| 9 \| *Tres*Man5A core – 5µl \| \| 10 \| LMW – 10µl \| \| 11 \| *Anig*Man5A – 5 µl \| \| 12 \| LMW – 10µl \| | 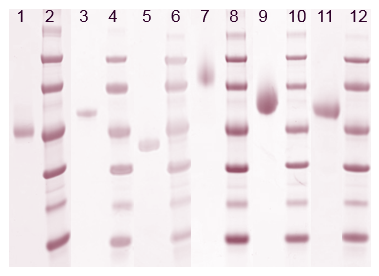 |  |
| --- | --- | --- | --- | --- | --- | --- | --- | --- | --- | --- | --- | --- | --- | --- | --- | --- | --- | --- | --- | --- | --- | --- | --- | --- | --- | --- | --- | --- |
| \| **Lane** \| **Sample** \| \| --- \| --- \| \| 1 \| *Cvir*Man26A – 5µl \| \| 2 \| LMW – 10µl \| \| 3 \| *Asti*Man26A – 5µl \| \| 4 \| LMW – 10µl \| \| 5 \| *Ypen*Man26A – 5µl \| \| 6 \| LMW – 10µl \| \| 7 \| *Wsp*Man26A – 5µl \| \| 8 \| Mark 12 – 10µl \| \| 9 \| *Ndes*Man26A – 5µl \| \| 10 \| Mark 12 – 10µl \| \| 11 \| *Mthe*Man5A – 5 µl \| \| 12 \| Mark 12 – 10µl \| | 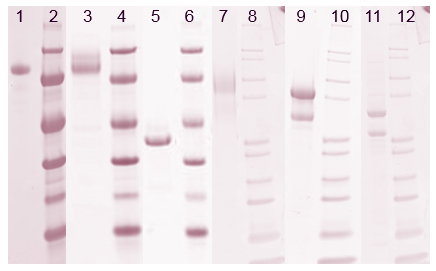 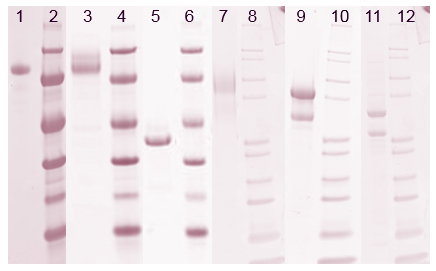 | 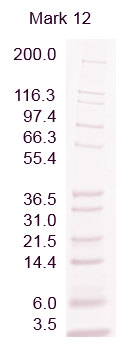 |

**Figure S3** SDS-PAGE gels of the purified enzymes. The protein concentration in the samples was 0.5 mg/ml. Prior to gel loading, samples were diluted 1:1 with loading mix. Loading mix was prepared as a 9:1 mix of Novex ® Tris-Glycine SDS Sample Buffer (2X) (Life Technologies) and Nupage ® Sample Reducing Agent (10X) (Life Technologies). Please consult the manuscript for enzyme abbreviations. Samples with *Ndes*Man26A and *Mthe*Man26A both contain molecules with and without the CBM35.
